# Evaluation of a commercial latex agglutination test for detecting rotavirus A and human adenovirus in children's stool specimens

**DOI:** 10.1002/jcla.23208

**Published:** 2020-01-13

**Authors:** Wenqing Xiang, Zhaoyang Peng, Jialu Xu, Hongqiang Shen, Wei Li

**Affiliations:** ^1^ Department of Clinical Laboratory The Children's Hospital Zhejiang University School of Medicine National Clinical Research Center For Child Health Hangzhou China; ^2^ Department of Neurology The Children's Hospital Zhejiang University School of Medicine National Clinical Research Center For Child Health Hangzhou China

**Keywords:** adenovirus, latex agglutination test, rotavirus A, RT‐qPCR, stool samples

## Abstract

**Objectives:**

Rotavirus A and human adenovirus are the two most common causes of infantile diarrhea; thus, it is of great importance to find out a rapid and accurate diagnostic method. This study aimed to evaluate the diagnostic significance of latex agglutination test for detection of rotavirus A and human adenovirus.

**Methods:**

A prospective study was conducted on 214 diarrhea children from September 2018 to March 2019 in our hospital. Fresh stool samples were collected for detection of rotavirus A and human adenovirus by latex agglutination test and quantitative reverse transcription polymerase chain reaction (RT‐qPCR). Then, the consistency of results detected by these two methods was analyzed．

**Results:**

With performing the latex agglutination test, it was revealed that positive rates for detecting rotavirus A virus and human adenovirus were 23.83% (51/214) and 25.24% (54/214), respectively. Meanwhile, results of RT‐qPCR showed that positive rates for detecting rotavirus A virus and human adenovirus were 58 (27.10%) and 59 (27.57%), respectively. Using RT‐qPCR as the gold standard, the sensitivity and specificity of the latex agglutination test for detecting rotavirus A were 81.03% and 97.44%, and the corresponding values for detecting human adenovirus were 76.27% and 94.19%, respectively.

**Conclusion:**

This latex agglutination test showed a satisfactory consistency with RT‐qPCR for detecting rotavirus A and human adenovirus. The mentioned commercial assay may be highly appropriate for rapid screening of rotavirus A and human adenovirus.

## 1 INTRODUCTION

Acute gastroenteritis (AGE) is a frequent infectious diarrhea disease, leading to hospitalization and eventually death in children aged <5 years old, especially in developing countries.^1^, ^2^, ^3^ It was reported that about 800 000 children who aged less than 5 years old annually died due to diarrhea. Several causes for AGE were reported, including unclean water, contaminated food, poor hygiene, and inadequate disposal of waste and feces, and the most general cause was found to be viral infection.^4^ Among numerous viruses, rotavirus (RV) and adenovirus (AdV) are the two most common causes for infantile diarrhea.^1^

In humans, RVs, which were firstly discovered in 1973 in duodenal biopsy of nine children who suffered from acute diarrhea, are non‐enveloped viruses of the Reoviridae family,^5^, ^6^ and group A (RVA) was found as the most common infectious variant in humans. AdV is a relatively large non‐enveloped dsDNA virus possessing a molecular weight of ~150 MDa.^7^ It has more than 80 different serotypes belonging to species A‐G,^8^ and they mainly infect humans through respiratory, ocular, and gastrointestinal. AdV has become the second principal reason for infantile diarrhea behind RV.^9^ Rapid and accurate diagnosis of AGE in early stage is of great importance for the treatment of AGE. Hence, a rapid and accurate diagnostic method for AGE is urgently required. Several techniques can be used to detect these two viruses, including scanning electron microscopy, polyacrylamide gel electrophoresis (PAGE), antigen detection assays, quantitative reverse transcription polymerase chain reaction (RT‐qPCR), enzyme‐linked immunoassay technology (ELISA), and virus isolation. However, it was previously noted that due complex operation, expensive equipment, etc, both scanning electron microscopy and PAGE are not highly appropriate to be used in the clinical practice. Moreover, the culture may lead to false‐negative results when fastidious or slowly growing viruses are involved.^10^, ^11^, ^12^, ^13^ At present, antigen detection assays and RT‐qPCR are the two most common detective methods worldwide.

In the present study, we utilized a commercial latex agglutination test kit, which is extensively used in numerous institutions across China. In clinical practice, RT‐qPCR is taken as a main method for detection of RV and AdV; however, it is extremely costly, making it inappropriate for early diagnosis. Hence, in the current research, RT‐qPCR was used as the gold standard to assess the capacity of the commercial LAT method for rapid detection of RV and AdV in outpatient children.

## 2 PATIENTS AND METHODS

### 2.1 Patients' selection

From September 2018 to March 2019, we selected patients who were admitted to the Children's Hospital of Zhejiang University, School of Medicine (Hangzhou, China), and met the following criteria: (a) children who aged <5 years old; (b) primary diagnosis of acute diarrhea with suspected virus infections; and (c) stool samples were simultaneously tested by latex agglutination test and RT‐qPCR during the study period. The design of this prospective study and the methods used were approved by the Ethics Committee of the Children's Hospital of Zhejiang University, School of Medicine.

### 2.2 RT‐qPCR for detection of rotavirus A and human adenovirus

Stool samples were collected and mixed with 1 mL normal saline. Then, 200 μL supernatant was separated from the mixture, which was centrifuged at 400 *g* at 20°C for 30 seconds, and DNA/RNA was extracted by NAE32 nucleic acid automatic extraction kit (DAAN Gene Co., Ltd.). The detection of rotavirus A and adenovirus was performed by SLAN 96P real‐time PCR System (HONGSHI Co., Ltd.) with a total volume of 25 μL. For each assay, negative and positive controls were implemented. FAM channel was used to detect rotavirus A, and HEX channel was employed to detect adenovirus. The amplification of RT‐qPCR products was conducted under the following conditions: for 20 minutes at 50°C; for 5 minutes at 95°C; five cycles for 10 seconds at 95°C, for 15 seconds at 55°C, and for 30 seconds at 72°C; and followed by 35 cycles for 10 seconds at 95°C and for 45 seconds at 60°C.

### 2.3 Latex agglutination test for detection of rotavirus A and human adenovirus

Latex agglutination test kit (Abon Biopharm Co., Ltd.) was used, which was approved by Food and Drug Administration (FDA) of China (Approval No. 20153402309). About 50 mg of stool samples was mixed with 1 mL sample extraction reagent. The well plate was composed of four major parts: (a) sample well plate; (b) control line; (c) rotavirus test line; and (d) adenovirus test line. Two drops (about 80 μL) of the mixture were also added into a sample well plate for 10‐20 minutes. If control line and test line (rotavirus) were both observed blue, the sample was determined as rotavirus positive. While control line was observed blue and test line (adenovirus) was observed red, the sample was determined as adenovirus positive.

### 2.4 Statistical analysis

The main purpose of the present study was to assess the sensitivity and specificity of the latex agglutination test compared with RT‐qPCR, which was considered as a standard method. The results were analyzed by using SPSS 20.0 software. The kappa test and Student's *t* test were used to determine statistical significance. *P* < .05 was considered statistically significant. The kappa (κ) value between 0.61 and 0.8 represented good agreement and between 0.81 and 1 reflected very good agreement.^14^, ^15^

## 3 RESULTS

To investigate the performance of commercial latex agglutination test kit, a total of 214 samples were collected in this study. As shown in Table 1, 58 (58/214, 27.10%) samples were detected positive for RV by the RT‐qPCR method, and 51 (51/214, 23.83%) samples were identified positive by the latex agglutination test. Among the samples, 47 (47/214, 21.96%) and 4 (4/214, 1.87%) samples were defined as true positive and false positive, respectively. In addition, 156 (156/214, 72.90%) samples were tested negative for RV by RT‐qPCR, and 163 (163/214, 76.17%) samples were found negative by latex agglutination test. Similarly, 152 (152/214, 71.03%) and 11 (11/214, 5.14%) samples were considered as true negative and false negative, respectively. According to statistical data, there was no remarkable difference between the two assays (*P* = .118, *χ* ^2^ = 2.4). Meanwhile, a very good agreement was observed between the two methods (κ value = 0.816). Considering RT‐qPCR as the gold standard, the sensitivity and specificity of the latex agglutination test were 81.03% and 97.44%, respectively. The positive prediction value and negative prediction value of the commercial assay were 92.16% and 93.25%, respectively.

**Table 1:** Comparing results of the latex agglutination test (LAT) with RT‐qPCR method for detection of RV

LAT assay	Real‐time PCR method		Total
	Positive	Negative	
Positive	47	4	51
Negative	11	152	163
Total	58	156	214

As shown in Table 2, 59 (59/214, 27.57%) samples were detected positive for AdV by RT‐qPCR method, and 54 (54/214, 25.23%) samples were identified positive by the latex agglutination test assay. Among the samples, 45 (45/214, 21.03%) samples were defined as true positive, and 9 (9/214, 4.21%) were defined as false positive. Additionally, 155 (155/214, 72.43%) samples were found negative for AdV by RT‐qPCR method, and 160 (160/214, 74.77%) samples were detected negative by latex agglutination test. Similarly, 146 (146/214, 68.22%) and 14 (14/214, 6.54%) samples were considered as true negative and false negative, respectively. There was no statistical significant difference between the two assays (*P* = .405, *χ* ^2^ = 0.696). A good agreement was also noted between these two methods for detecting AdV (κ value = 0.724). Considering RT‐qPCR as the gold standard, the sensitivity and specificity of the latex agglutination test were 76.27% and 94.19%, respectively. The positive prediction value and negative prediction value of the commercial assay were 83.33% and 91.25%, respectively.

**Table 2:** Comparing results of latex agglutination test (LAT) with RT‐qPCR method for detection of AdV

LAT assay	Real‐time PCR method		Total
	Positive	Negative	
Positive	45	9	54
Negative	14	146	160
Total	59	155	214

## 4 DISCUSSION

Diarrhea is a common and frequent disease in infants and young children,^16^ and its incidence rate ranks the second to the upper respiratory tract infection. Recently, due to improved hygienic conditions and the widespread use of antibiotics, the incidence of diarrhea caused by bacterial has dropped. However, the morbidity of diarrhea caused by virus has increased, and the most common viruses were RV and AdV. A rapid antigen test is helpful for screening the RV and AdV; however, the values of sensitivity of different methods are quite different. In the present study, a commercial kit was tested, which is widely engaged in more than 200 hospitals in China. The mentioned kit is highly convenient for operating without instrument. Compared with RT‐qPCR, the turnaround time (TAT) of LAT assay was found to be shorter. When samples were collected, RT‐qPCR method takes about 3 hours to finish, while the LAT assay only requires about 15‐20 minutes. Although Alere i assay (Isothermal amplification of nucleic acid) can provide results within 30 minutes, this technology is now mainly used for respiratory virus detection, such as influenza A virus, influenza B virus, respiratory syncytial virus, and it has not been used for rotavirus and adenovirus detection.^17^ Moreover, this method has not been applied in China.

Furthermore, the LAT assay for detection of RV and AdV is a lower cost method (about 9 dollars per test), while RT‐qPCR is relatively costly (about 23 dollars per test). Because of rapid detection and low cost, it is appropriate for detection of AdV and RV in outpatients. However, RT‐qPCR is suitable for detecting RV and AdV in inpatients. In this study, if results of LAT assay were inconsistent with RT‐qPCR, we confirmed as false positives or false negatives.

According to a previous study, virus is further prevalent in autumn and winter seasons, because viruses may be more stable at a low temperature.^18^ In the present study, we successfully enrolled 214 patients who were admitted to our hospital in the aforementioned seasons. Our data showed that positive rates of RV were 23.83% and 27.10% for latex agglutination test and RT‐qPCR, respectively. Moreover, the positive rates of AdV were 25.23% and 27.57% for latex agglutination test and RT‐qPCR, respectively. Both of the positive rates were relatively remarkable, while previous studies reported different prevalence rates.^19^, ^20^, ^21^ The results of the current research indicated that there was no significant difference (*P* > .05) between the latex agglutination test and RT‐qPCR in the detection of RV and AdV. Meanwhile, it was noted that there was a good agreement in detection of RV and AdV between these two methods. To evaluate the performance of the commercial LAT assay kit, we used RT‐qPCR as the gold standard. The findings unveiled sensitivity and specificity of RV were 81.03% and 97.44%, respectively. Cevenini et al^22^ reported that the sensitivity of LAT for detecting RV outperformed immunofluorescence, while the specificity was lower than other three methods, such as scanning electron microscopy, immunofluorescence, and ELISA. Another study demonstrated a sensitivity of 85.9% and a specificity of 97.7% for detecting RVA.^23^ The findings of our present research disclosed that the sensitivity and the specificity for detecting AdV were 76.27% and 94.19%, respectively. Although the current study showed that there was no significant difference between the two assays, 11 RV samples and 14 AdV samples were positive for RT‐qPCR and negative for LAT. This may due to low sensitivity of LAT while cycle threshold (Ct) value of these samples was above 30 by RT‐qPCR. We also found 4 RV samples and 9 AdV samples were negative for RT‐qPCR and positive for LAT. These results may be considered as false positive which indicated the specificity of LAT assay was not high enough. The RT‐qPCR may be further appropriate for confirming the viruses, while LAT is highly recommended for screening. This research was a case‐control study, and the coincidence rate of the positive rate between the two assays was slightly more than daily monitoring. In the next study, we will expand the sample size to find out differences between the two methods. Moreover, we found that there were two cross‐reactional samples, indicating that the test line of RV was red and the test line of AdV was blue. There were several reasons can cause cross‐reaction, but the most probably reason may be the fecal samples with too much blood, which was caused by multiple bowel movements. In clinical practice, special attention is needed for these cases.

## 5 CONCLUSIONS

The proposed commercial latex agglutination test is a low‐cost screening method for identification of rotavirus and adenovirus with a middle level of sensitivity and specificity. However, the mentioned method is not highly appropriate for accurate diagnosis of rotavirus and adenovirus in hospitalized children. In the future study, we will further evaluate fast diagnostic methods with consideration of lager sample size.

## CONFLICT OF INTEREST

The authors declare that there is no conflict of interest.

## References

[1] Wu BS , Huang ZM , Weng YW , et al. Prevalence and genotypes of rotavirus A and human adenovirus among hospitalized children with acute gastroenteritis in Fujian, China, 2009–2017. Biomed Environ Sci. 2019;32(3):210‐214. 30987695 10.3967/bes2019.028

[2] Enweronu‐Laryea CC , Sagoe KW , Glover‐Addy H , et al. Prevalence of severe acute rotavirus gastroenteritis and intussusceptions in Ghanaian children under 5 years of age. J Infect Dev Ctries. 2012;6:148‐155. 22337844 10.3855/jidc.1667

[3] Willame C , Noordegraaf‐Schouten MV , Gvozdenović E , et al. Effectiveness of the oral human attenuated rotavirus vaccine: a systematic review and meta‐analysis 2006–2016. Open Forum Infect Dis. 2018; 5(11):ofy292. 30539038 10.1093/ofid/ofy292 PMC6284461

[4] Shadi TN , Seyed Reza M , Amir G , Seyed MH . Human rotavirus in Iran; molecular epidemiology, genetic diversity and recent updates on vaccine advances. Spring. 2019;12(2):98‐109. PMC6536013 31191833

[5] Sadiq A , Bostan N , Yinda KC , et al. Rotavirus: genetics, pathogenesis and vaccine advances. Rev Med Virol. 2018;28(6):e2003. 30156344 10.1002/rmv.2003

[6] Afchangi A , Jalilvand S , Mohajel N , et al. Rotavirus VP6 as a potential vaccine candidate. Rev Med Virol. 2019;29(2):e2027. 30614135 10.1002/rmv.2027

[7] Smith JG , Wiethoff CM , Stewart PL , Nemerow GR . Adenovirus. Curr Top Microbiol Immunol. 2010;343:195‐224. 20376613 10.1007/82_2010_16 PMC3093298

[8] Urs FG , Justin WF . Adenovirus entry: from infection to immunity. Annu Rev Virol. 2019; 6(1): 177-197. 31283442 10.1146/annurev-virology-092818-015550

[9] Ozsri T , Bora G , Kaya B , et al. The prevalence of rotavirus and adenovirus in the childhood gastroenteritis. Jundishapur J Microbiol. 2016;9(6):e34867. 27635215 10.5812/jjm.34867 PMC5012192

[10] Li YF , Fu ZP , Li G , et al. Development of a quantitative ELISA for detection of rotavirus antigen. Chin J Viral Dis. 2016;6(4):288‐293.

[11] Unal N , Yanik K , Aydogdu S , et al. An evaluation of rotavirus and adenovirus antigens by the immunochromatographic method in samples with an initial diagnosis of acute gastroenteritis. Acta Medica Mediterranea. 2016;32(1):81‐85.

[12] Steele JC Jr . Rotavirus. Clin Lab Med. 1999;19(3):691‐703. 10549433

[13] Tolba M , Ahmed MU , Tlili C , et al. A bacteriophage endolysin‐based electrochemical impedance biosensor for the rapid detection of Listeria cells. Analyst (Lond). 2012;137(24):5749‐5756. 23085745 10.1039/c2an35988j

[14] Yun SG , Kim MY , Choi JM , et al. Comparison of three multiplexPCR assays for detection of respiratory viruses: Anyplex II RV16, AdvanSure RV, and Real‐Q RV. J Clin Lab Anal. 2018;32:e22230. 10.1002/jcla.22230 PMC5836940 28397965

[15] Altman DG . Practical Statistics for Medical Research. London: Chapman & Hall/CRC; 1991.

[16] Das JK , Salam RA , Bhutta ZA . Global burden of childhood diarrhea and interventions. Curr Opin Infect Dis. 2014;27(5):451‐455. 25101554 10.1097/QCO.0000000000000096

[17] Schnee SV , Pfeil J , Ihling CM , et al. Performance of the Alere i RSV assay for point‐of‐care detection of respiratory syncytial virus in children. BMC Infect Dis. 2017;17(1):767. 29237419 10.1186/s12879-017-2855-1 PMC5729395

[18] Zaraket H , Charide R , Kreidieh K , et al. Update on the epidemiology of rotavirus in the Middle East and North Africa. Vaccine. 2017;5(45):6047‐6058. 10.1016/j.vaccine.2017.09.067 28986034

[19] Liu L , Qian Y , Zhang Y , et al. Epidemiological aspects of rotavirus and adenovirus in hospitalized children with diarrhea: a 5‐year survey in Beijing. BMC Infect Dis. 2016;16(1):508. 27663354 10.1186/s12879-016-1829-z PMC5035450

[20] Ye HY , Zhou FM , Cui DW , et al. Distribution and clinical features of gastrointestinal virus infection in infants with acute diarrhea. Chin J Clin Infect Dis. 2013;6(6):335‐338.

[21] Gao J , Zhang HY , Zhang GL , et al. Epidemiological characteristics of infantile rotavirus diarrhea in Harbin in 2014. J Harbin Med Univ. 2016;50(2):159‐165.

[22] Cevenini R , Rumpianesi F , Mazzaracchio R , et al. Evaluation of a new latex agglutination test for detecting human rotavirus in faeces. J Infect. 1983;7(2):130‐133. 6315827 10.1016/s0163-4453(83)90527-3

[23] Dusetty P , Velázquez FR , Gutiérrez‐Escolano AL , Ludert JE . Evaluation of the second generation of a commercial latex agglutination test for the detection of rotavirus antigens in fecal samples. J Clin Virol. 2013;57(1):88‐90. 23403240 10.1016/j.jcv.2013.01.011

